# The Impact of Age on the Association Between Physical Activity and White Matter Integrity in Cognitively Healthy Older Adults

**DOI:** 10.3389/fnagi.2020.579470

**Published:** 2020-11-05

**Authors:** Dominik Wolf, Florian U. Fischer, David Riedel, Kristel Knaepen, Bianca Kollmann, Merve Kocabayoglu, Katharina Brüggen, Stefan Teipel, Oliver Tüscher, Harald Binder, Andreas Mierau, Andreas Fellgiebel

**Affiliations:** ^1^Department of Psychiatry and Psychotherapy, University Medical Center Mainz, Mainz, Germany; ^2^Center for Mental Health in Old Age & the German AgeGain Study Group, Mainz, Germany; ^3^German Sport University Cologne, Institute of Movement and Neurosciences, Cologne, Germany; ^4^Leibnitz Institute for Resilience Research (LIR), Mainz, Germany; ^5^German Center for Neurodegenerative Diseases (DZNE), Rostock, Germany; ^6^Department for Psychosomatic and Psychotherapeutical Medicine, University Hospital Rostock, Rostock, Germany; ^7^Institute of Medical Biometry and Statistics (IMBI), University of Freiburg, Freiburg, Germany; ^8^Department of Exercise and Sport Science, LUNEX International University of Health, Exercise and Sports, Differdange, Luxembourg

**Keywords:** healthy aging, physical activity, actigraphy, white matter integrity, cognition

## Abstract

Cognition emerges from coordinated processing among distributed cortical brain regions, enabled through interconnected white matter networks. Cortical disconnection caused by age-related decline in white matter integrity (WMI) is likely to contribute to age-related cognitive decline. Physical activity (PA) has been suggested to have beneficial effects on white matter structure. However, its potential to counteract age-related decline in WMI is not yet well established. The present explorative study analyzed if PA was associated with WMI in cognitively healthy older adults and if this association was modulated by age. Forty-four cognitively healthy older individuals (aged 60–88 years) with diffusion-tensor imaging (DTI) and PA measurements were included from the AgeGain study. Voxelwise analysis using Tract-Based Spatial Statistics (TBSS) demonstrated that PA was associated with WMI in older adults. However, results emphasized that this association was restricted to high age. The association between PA and WMI was found in widespread white matter regions suggesting a global rather than a regional effect. Supplementary analyses demonstrated an association between the integrity of these regions and the performance in memory [verbal learning and memory test (VLMT)] and executive functioning (Tower of London).Results of the present explorative study support the assumption that PA is associated with WMI in older adults. However, results emphasize that this association is restricted to high age. Since cognitive decline in the elderly is typically most pronounced in later stages of aging, PA qualifies as a promising tool to foster resilience against age-related cognitive decline, *via* the preservation of the integrity of the brains WM.

## Introduction

Normal aging is characterized by a cognitive decline in several cognitive domains, including memory, information processing speed, reasoning, spatial orientation, and numeric abilities (Salthouse, 2010; Park and Schwarz, 2012). However, the interindividual variability of age-related cognitive changes is high, ranging from clinically apparent reduction to far-reaching preservation of cognitive functioning (Wilson et al., 2002). Cognitive health has been quoted as a major factor for life quality in the elderly and contributes greatly to late-life functioning and independence (Depp and Jeste, 2006; Reichstadt et al., 2007). This significance of cognitive health highlights the importance of a profound understanding of the preservation of cognitive functioning in aging.

Cognition relies on the coordinated processing among distributed cortical brain regions, enabled through interconnected white matter networks. Cortical disconnection has been shown to lead to cognitive dysfunction (Catani and ffytche, 2005; Filley, 2005). Cortical disconnection is also regarded as a key concept in age-related cognitive decline since several diffusion-tensor imaging (DTI) studies indicate that the integrity of white matter networks typically declines in healthy aging and that cognitive abilities in aging are associated with the integrity of white matter networks (Madden et al., 2009, 2012; Bennett and Madden, 2014). In line with the variance in age-related cognitive decline, studies have also highlighted substantial between-subject variance in age-related changes in white matter integrity (WMI; Madden et al., 2012; Bennett and Madden, 2014; Sexton et al., 2014). Thus, the preservation of WMI is a promising approach to prevent cortical disconnection and maintain cognitive abilities within the aging process.

Concerning this, higher levels of physical activity (PA) have been shown to have beneficial effects on various structural properties in the brain in older adults, especially on gray matter volume (Bherer et al., 2013; Erickson et al., 2014). A growing number of studies now focuses on the investigation of the effects of PA on white matter properties (Sexton et al., 2016). In addition to repeated findings of an association between PA and white matter volume as well as white matter lesions (Sexton et al., 2016), many studies reported a positive effect of PA on WMI (Gow et al., 2012; Johnson et al., 2012; Liu et al., 2012; Tseng et al., 2013a). PA thus qualifies as a promising tool to counteract the age-related decline in WMI and to preserve cognition functioning *via* the association with WMI. However, several negative findings on the association between PA and WMI have also been published (Voss et al., 2013; Burzynska et al., 2014a; Tian et al., 2014). Hence, the effect of PA on WMI has not yet been well established.

It has been suggested that greater levels of PA may particularly be beneficial in later life since a decline in white matter microstructure is most pronounced in high age. This may contribute to the heterogeneity in study results and highlights the necessity of a better understanding of the trajectory of the association between PA and WMI with age (Sexton et al., 2016). The present explorative study aimed at investigating if PA is associated with WMI in healthy elderly and if this association is modulated by age. In supplementary analyses, the study also analyzed if the integrity of white matter regions that were associated with PA was also related to cognitive performance.

## Materials and Methods

### Data Source and Subjects

Data used in the present article were obtained from the AgeGain study (Wolf et al., 2018). The multicenter trial AgeGain primarily aims at the investigation of mechanisms and modulators of transfer of cognitive training gains in cognitively healthy elderly. The main exclusion criteria were current (or history of) psychiatric, cognitive, neurological, or cardiovascular diseases, brain lesions, or secondary disorders restricting individuals’ physical capacity (i.e., chronic obstructive pulmonary disease). A full description of AgeGain, including details on the comprehensive database of the study as well as detailed information on in-and exclusion criteria, can be found in the study protocol (Wolf et al., 2018). Data were taken from an interim subset of data. Baseline DTI-scans, actigraphy-based measures of PA, and cognitive data of 44 cognitively healthy older adults (age range: 60–88 years) were included.

### Physical Activity

Physical activity was assessed using triaxial accelerometers (GENEActiv, ActivInsights Limited, Kimbolton, Cambridgeshire, UK). Participants were asked to wear the accelerometers on their non-dominant wrists 24 h a day. The devices were set to collect data at 100 Hz for seven consecutive days. The extracted raw data were processed using R package GGIR version 1.9-1 (Migueles et al., 2019).

The analyses included autocalibration (Van Hees et al., 2014), detection and imputation of non-wear time as well as the extraction of the mean Euclidean Norm minus 1 (ENMO) for the acquisition period of seven consecutive days (Van Hees et al., 2013). The ENMO metric is computed from the resultant vector of the measured orthogonal acceleration and adjusts for gravity *via* subtracting a fixed offset of one gravitational unit from the Euclidean Norm of the three raw acceleration signals. The ENMO metric reflects the mean overall physical activity (Migueles et al., 2019) and correlates with physical activity-related energy expenditure (Van Hees et al., 2013). Its application has become common even in large scale studies (Doherty et al., 2017; Menai et al., 2017; Sanders et al., 2019).

### Neuropsychology

The performance of the cognitive domains memory, executive function, and information processing speed was assessed. All three domains are typically affected in aging.

Memory performance was measured using the verbal learning and memory test (VLMT), a German version of the auditory verbal learning test, which requires learning of a list of 15 words in five consecutive trials, free recall of these words after each trial, and recognition after 20 min. The performance was rated according to the sum of correct remembered words of all five trials (VLMT 1–5) and correct recognized words after 20 min (VLMT recognition).

Executive function was measured using the Tower of London task (ToL), which is a measure of planning and problem-solving. The ToL test consists of a set of three pegs on a wooden base and three differently colored balls which can be moved on the pegs. A start configuration of the balls has to be transformed into a given goal configuration while considering the following rules: only one ball can be moved at a time, a ball is not allowed to be placed next to a peg, and only the topmost ball of each peg can be moved. One peg can hold a maximum of three balls, one takes a maximum of two balls and the third takes only one ball. Subjects are instructed to solve a given problem in the minimum number of moves. Trials included problems, where three- to six moves were required to solve the task. The performance was rated based on the number of correctly solved problems.

Information processing speed was assessed by the trail-making-test A (TMT-A). The test requires an individual to draw lines sequentially connecting 25 encircled numbers distributed on a sheet of paper. Participants were instructed to complete the task as quickly and accurately as possible. In case of an error, participants were instructed to return to the circle where the error occurred and continue. The performance was rated according to the response time.

### Imaging Data Acquisition and Processing

DWI imaging data were acquired on Siemens Magnetom Trio and Verio 3T scanners using a multiband sequence with a voxel size of 2 × 2 × 2 mm^3^, 64 diffusion gradients, a multiband factor of 3, and *b*-values of 0 and 2,000 s/mm^2^. For distortion correction purposes, an additional *b* = 0 image with inverted phase encoding, i.e., posterior-anterior, was acquired. DWI data were corrected for eddy current and susceptibility induced artifacts and distortions using “FSL” (Jenkinson et al., 2012) and the included software eddy (Andersson and Sotiropoulos, 2016) as well as top-up (Andersson et al., 2003), which estimates a susceptibility induced off-resonance field using the phase inverted *b* = 0 image. Subsequently, we used “mrtrix” (Tournier et al., 2019) to fit a diffusion tensor to the data with a weighted linear least squares estimator. Finally, fractional anisotropy (FA) and mean diffusivity (MD) images were calculated from the eigensystem of the estimated diffusion tensors.

### Data Analyses

We applied whole-brain voxel-wise regression analyses using Tract-Based Spatial Statistics (TBSS; Smith et al., 2006) to investigate the association between WMI (as quantified by FA and MD) and physical activity. TBSS preprocessing included the following steps: (i) nonlinear registration to the FMRIB58_FA template of all subjects’ FA data using FMRIB’s nonlinear image registration tool (Rueckert et al., 1999); (ii) creation and thinning of a mean FA image with a threshold of 0.2 to obtain a mean FA skeleton that represents the centers of white matter trajectories; and (iii) projection of each subjects’ aligned data onto the skeleton. To achieve skeletonized MD data, the nonlinear warps and skeleton projection vectors of the FA images were applied to the MD data. Regression analyses were performed using the randomize tool, which tested the *t*-value at each voxel against a null distribution that was obtained from 5,000 random permutations. To minimize the probability of false-positive voxels, analyses were corrected for multiple comparisons across voxels using the threshold-free cluster-enhancement option.

Initially, we tested the general association between WMI and physical activity (mean ENMO). Age was added as a control variable since it is associated with both, WMI and physical activity (Bennett and Madden, 2014; McPhee et al., 2016). Thereafter, we added the interaction term *PA × age* to the regression model to investigate if the association between physical activity and WMI was modulated by age. Significant regions on the TBSS skeleton were extracted as masks (separately for each regression model) and mean WMI was calculated for each mask. Mean WMI values were then used to characterize potential interaction effects.

Moreover, in explorative analyses, the association between mean WMI values and cognitive performance was investigated using robust regression analyses (based on an MM estimator). Age was added as a control variable since it is known to be associated with WMI and cognition (Salthouse, 2010; Bennett and Madden, 2014). Statistical analyses outside the TBSS framework were carried out using the statistical software package R 3.0.2.

## Results

### Descriptive Characteristics

Descriptive characteristics of the study group are summarized in Table 1. The mean age of the study group was 69.07 (SD, 7.3) years, 29 individuals (65.9%) were female. Participants were highly educated with a mean educational attainment of 15.43 (SD, 2.2) years. There was no difference in educational attainment between males and females. Education was not related to age. Age was negatively associated with PA (*r_Perason_* = −0.32, *p* = 0.04). Education and gender were not associated with PA. Moreover, age was negatively associated with short-term memory (*r_Pearson_* = −0.31, *p* = 0.04) and executive functioning (*r_Pearson_* = −0.34, *p* = 0.02), but not with long-term memory and information processing speed. Education and gender were not associated with any cognitive measure.

**Table 1:** Descriptive and cognitive characteristics.

	Study Group (*n* = 44)
Demographic characteristics	
Age, mean (SD), years	69.07 (7.3)
Age range, years	60–88
Woman (%)	29 (65.9)
Education, mean (SD), years	15.43 (2.2)
Physical activity (ENMO mg/day)	26.99 (6.6)
Cognitive characteristics	
VLMT 1–5 (number of correct responses)	50.75 (7.7)
VLMT recognition (number of correct responses)	10.00 (2.8)
ToL (number of correctly solved problem)	15.82 (2.1)
TMT-A (seconds)	37.19 (9.7)

*Abbreviations: ENMO, Euclidean Norm minus 1 in milligravity units per day; VLMT, verbal learning and memory test; ToL, tower of London; TMT-A, trail-making test A; y, years*.

### Association Between WMI and PA

**Figure 1:**
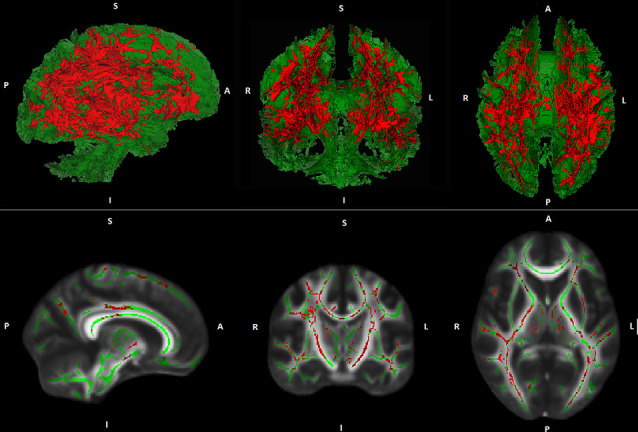
Illustration of Tract-based spatial statistic (TBSS) results. Interaction effects (red) between age and physical activity (PA) on white matter mean diffusivity (MD) are projected on a cerebral white matter skeleton (green; 3D above, 2D below).

Whole-brain voxel-wise TBSS regression analyses demonstrated no general association between PA and FA- or MD values. However, regression analyses demonstrated an interaction between PA and age on MD. This result indicates that the association between PA and MD is age-dependent. The interaction was found in widespread white matter regions suggesting a global rather than a regional effect (see Figure 1). A comparable interaction pattern between PA and age on FA values has been observed. However, results were less strong and did not reach statistical significance at *p* ≤ 0.05, corrected for multiple comparisons.

**Figure 2:**
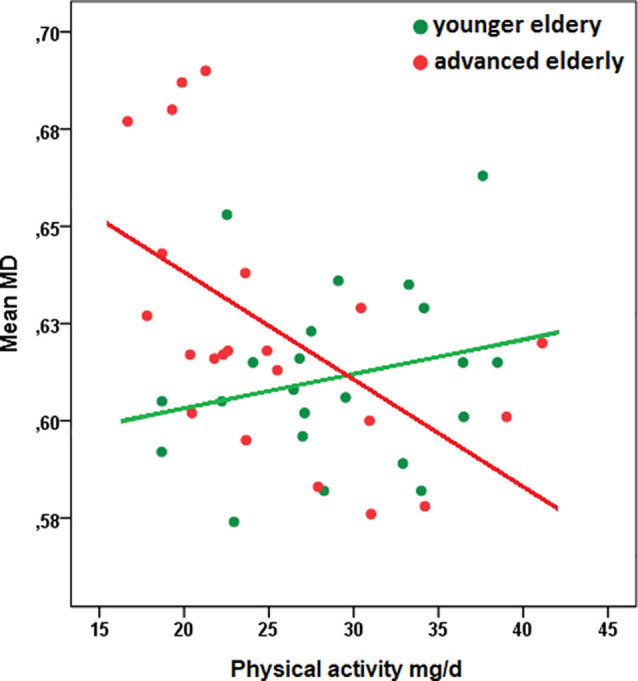
Scatterplot of the relationship between physical activity and mean MD of white matter regions that were associated with the interaction term *PA × age*, separated for younger- and advanced elderly defined by a median split (green: younger elderly, *n* = 22; red: advanced elderly, *n* = 22). The median split has been applied only for illustration purposes. All statistical analyses were calculated based on continuous data. Descriptive characteristics of subgroups: younger elderly, mean age in years (SD): 64.5 (1.7), woman (%): 13 (59.1), education in years: 15.5 (1.5), physical activity in mg/d: 28.8 (6.0); advanced elderly: mean age (SD): 74.6 (6.5), woman (%): 16 (72.7), education in years (SD): 15.4 (2.8), physical activity in mg/d: 25.2 (6.7); Pearson correlation coefficient between PA and mean MD in subgroups: younger elderly, *r* = 0.24, *p* = 0.28; advanced elderly, *r* = −.55, *p* = 0.01. Abbreviations: MD, mean diffusivity; ENMO, Euclidean Norm minus 1 in milligravity units per day.

Regions demonstrating significant interaction effects between PA and age on MD have been extracted as a separate mask and mean MD values within this mask have been calculated. A scatter plot to characterize the observed interaction indicated a stronger association between PA and mean MD with increasing age (see Figure 2; of note: for convenient visualization of the interaction effect, the association between PA and MD values has been plotted separately for younger- and advanced elderly, as defined by a median split. The median split has only been applied for illustration purposes. All statistical analyses were calculated based on continuous data).

### Association Between Cognition and WMI of Regions Associated With PA

Supplementary robust regression analyses were applied to investigate the association between cognitive performance and mean MD values of the extracted mask (with age added as a covariate of no interest). A negative association could be observed between mean MD values and executive functioning (ToL: *ß* = −0.29, *p* = 0.03). Negative associations could also be observed between mean MD values and short- as well as long term memory. However, these associations did not reach statistical significance (VLMT 1–5: *ß* = −0.34, *p* = 0.09; VLMT recognition: *ß* = −0.18, *p* = 0.07). Information processing speed was not related to mean MD of the extracted mask (TMT-A: *ß* = −0.07, *p* = 0.59).

## Discussion

The present explorative study aimed at increasing the understanding of the association between PA and WMI in cognitively healthy elderly. The study indicates that PA is associated with WMI in older adults. However, results emphasize that this association is restricted to higher age.

Over the last years, an increasing number of studies investigated the association between PA and WM measures in cognitively healthy elderly. Many of these studies found that higher levels of PA were related to greater WM volumes (Colcombe et al., 2006; Ho et al., 2011; Gow et al., 2012; Benedict et al., 2013; Tseng et al., 2013b) as well as reduced volume and severity of WM lesions (Sen et al., 2012; Wirth et al., 2014). Although some negative findings have also been published, meta-analyses could confirm the described results (Torres et al., 2015; Sexton et al., 2016). Studies on the relation between PA and WM microstructure are less frequent. In contrast to the mostly homogeneous results on the association between PA and WM volume as well as WM lesions, results on the relationship between PA and WM microstructure are heterogeneous. On the one hand, studies found positive associations between PA and global as well as regional WM FA in cognitively healthy elderly (Gow et al., 2012; Johnson et al., 2012; Liu et al., 2012; Tseng et al., 2013a). Moreover, negative associations between PA and regional WM MD have been reported (Tseng et al., 2013a). On the other hand, several studies did not find any correlations between PA and measures of WM microstructure (Voss et al., 2013; Burzynska et al., 2014b; Tian et al., 2014). Since advancing age is associated with accelerated decline in WM microstructure, it has been suggested that the association between PA and WM microstructure may be more pronounced in (or restricted to) advanced stages of aging (Sexton et al., 2016). The current study supports this hypothesis and thus provides important information for a better understanding of the largely unknown trajectory of the relationship between PA and WM microstructure in the process of aging. Of note, our results imply that the association between PA and WM microstructure is rather global instead of being locally restricted (see Figure 1).

PA as assessed using triaxial accelerometers over several days in everyday life can be seen as a measure of general activity level in the sense of a trait marker. The association between the general PA level and WMI may be mediated by various neurobiological mechanisms. First, a higher level of PA leads to a persistent upregulation of neurotrophic factors, especially of brain-derived neurotrophic factor (BDNF; Huang et al., 2014). BDNF has been shown to support synaptic plasticity and normal axonal pruning (Cao et al., 2007; Singh et al., 2008). Moreover, it has been observed that BDNF has a neuroprotective influence on white matter in both rodents and humans (Husson et al., 2005; Weinstock-Guttman et al., 2007). The association between PA and WMI may thus be mediated by upregulated BDNF in response to PA. Second, there is some evidence that suggests that higher levels of PA may lead to the proliferation of oligodendrocyte progenitor cells, which continue to form new oligodendrocytes and allow for continued myelination (Krityakiarana et al., 2010). Thus, the extent of axonal myelination and therewith the integrity of WM pathways may alter in response to PA, mediated by increased oligodendrocyte proliferation. Third, higher levels of PA have been shown to have beneficial effects on the vascular system, including the preservation of arterial elasticity and wall integrity, as well as a reduction in arterial stiffness and blood pressure (McDonnell et al., 2013). Moreover, some animal studies reported increased capillary density in the consequence of PA (Swain et al., 2003; Ding et al., 2006). Improved vascular health caused by PA may thus contribute to increased WMI *via* improved oxygen and nutrient delivery (Sexton et al., 2016). Although positive associations between PA and brain structure have typically been interpreted as the result of positive effects of PA on brain structure it is also possible that better cerebral function, especially in old age, may lead to higher PA. A better cerebral function in old age may be associated with a more independent lifestyle, which in turn might be associated with higher PA.

Although a comparable interaction pattern between PA and age on FA and MD values has been observed, results between PA and FA values were less strong and did not reach statistical significance (corrected for multiple comparisons). Fa reflects non-isotropic diffusion. Although FA is very sensitive to microstructural changes, it is not thought to be specific to any particular tissue characteristic (Uddin et al., 2019). MD is a measure of the average magnitude of water diffusion and is used to infer the overall density of tissue barriers. It is thought to reflect the density of synapses, capillaries, and macromolecular proteins as well as changes in cell proliferation and the shapes of neurons or glia (Uddin et al., 2019). Many of these microstructural properties are related to PA (as described above), which might explain the stronger interaction pattern between PA and MD values compared to FA values.

PA has repeatedly been shown to be associated with cognitive functioning in older adults and with a reduced risk for developing Alzheimer’s disease (Kramer et al., 2006; Hillman et al., 2008; Buchman et al., 2012). A high level of PA thus qualifies as an important lifestyle factor to increase resilience against age-related cognitive decline. The neurobiological mechanisms underlying the association between PA and cognition in the elderly are not fully understood. It has been discussed that the positive effects of PA on cognition may be mediated by changes in brain structure and function induced by PA (Kramer and Erickson, 2007; Hillman et al., 2008). However, the role of WMI in the association between PA and cognition is not yet clear. Exploratory regression analyses of the current study demonstrated that WMI of regions that were associated with PA was also related to memory and executive functioning (see “Results” section). These results suggest that changes in WMI caused by PA may mediate the association between PA and cognition in older adults. This assumption is in line with the fact that cognition relies on coordinated processing among distributed cortical brain regions, which is enabled through interconnected white matter networks and that cortical disconnection has been shown to lead to cognitive dysfunction (Catani and ffytche, 2005; Filley, 2005). However, specific mediation analyses in larger study samples are necessary to confirm and specify the role of WMI in the association between PA and cognition in older adults.

An advantage of this study is the assessment of the general PA level using triaxial accelerometers. Triaxial accelerometers allow an objective measurement of PA that does not require any input from the participant and that is not associated with disadvantages typically seen in commonly used self-reports, such as recall bias and fluctuations in answers caused by health status, depression, anxiety, or cognitive ability (Rikli, 2000). Moreover, in comparison to self-reports, stronger relationships between objective accelerometer data and health characteristics, such as obesity or depressive symptoms, have been shown (Koolhaas et al., 2017; Guo et al., 2019). These results imply a higher validity of accelerometer data compared to self-reported data. Triaxial accelerometers are particularly appropriate for the assessment of PA in older adults since bias to the subjective recall of past events is typically more pronounced in advanced age (Barnett et al., 2016).

### Methodological Considerations/Limitations

The study has several limitations. First, most of the study participants were highly educated, which might have been associated with a more active lifestyle. This in turn might be accompanied by a small variance in the amount of PA. Moreover, PA-related variations in brain structure might have been underestimated given a potentially homogeneous high level of PA within the study group. Future studies should confirm the observed results in subjects with a broad range of educational attainment and should investigate if the observed association between PA and WMI in older adults is independent of the general activity level. Second, the cross-sectional nature of the analysis does not allow the inference of causality. Third, due to the limited sample size, analyses might have been sensitive to type 2 errors. A power calculation has not been applied since this study is explorative. Future studies should confirm the findings of this study based on a larger study cohort.

## Conclusion

Cortical disconnection caused by an age-related decline in WMI is likely to contribute to age-related cognitive decline. Hence, the prevention of white matter degeneration in aging is a promising approach to preserve cognitive functioning and late-life independency. The results of the present study demonstrate that PA is associated with WMI in older adults. Of note, results emphasize that this association is restricted to advanced stages of aging. Moreover, exploratory analyses demonstrated that WMI of regions that were associated with PA was also related to memory and executive functioning. Since cognitive decline in older adults is typically most pronounced in later stages of aging, PA qualifies as a promising tool to foster resilience against age-related cognitive decline, *via* the preservation of the integrity of the brains WM.

## Data Availability Statement

The datasets generated for this study are available on request to the corresponding author.

## Ethics Statement

The studies involving human participants were reviewed and approved by Ethics commissions of the Landesärztekammer Rheinland-Pfalz Ethics commissions of the Cologne University’s Faculty of Medicine and Ethics commissions of the Rostock University’s Faculty of Medicine. The patients/participants provided their written informed consent to participate in this study.

## Author Contributions

DW: study design, data analyses, interpretation, and preparation of the manuscript. FF: data analyses and interpretation. DR: data acquisition and data analyses. KK, BK, MK, and KB: data acquisition. ST and OT: revision of the manuscript. HB: statistical support. AF: study design, interpretation, and revision of the manuscript. All authors contributed to the article and approved the submitted version.

## Conflict of Interest

The authors declare that the research was conducted in the absence of any commercial or financial relationships that could be construed as a potential conflict of interest. The reviewer GG-E declared a shared affiliation, with no collaboration, with several of the authors, DW, FF, BK, MK, OT, AF, to the handling editor at the time of review.
